# Epidemiological insights and survival patterns in neuroendocrine neoplasia: a geographic perspective

**DOI:** 10.1530/EO-25-0039

**Published:** 2025-08-05

**Authors:** Jessica Mangion, Josanne Vassallo, Mark Gruppetta

**Affiliations:** ^1^ Department of Medicine, Faculty of Medicine and Surgery, University of Malta, Mater Dei Hospital, Msida, Malta; ^2^ Neuroendocrine Clinic, Department of Medicine, Mater Dei Hospital, Msida, Malta

**Keywords:** epidemiology, survival, neuroendocrine neoplasia, gastroenteropancreatic, bronchopulmonary, America, Europe, Asia, Australia

## Abstract

Neuroendocrine neoplasms, though rare, have shown marked increases in global incidence and prevalence over the past decade, as demonstrated by cancer registry data and studies from specialised tertiary centres. However, it remains unclear whether this represents a true increase in incidence or enhanced detection capabilities across various countries. This review aims to analyse and discuss recently published data on the worldwide epidemiology of gastroenteropancreatic NEN, and bronchopulmonary NEN and explore potential trends and differences in incidence rates between large and small nations according to demographics, primary sites, grade and stage. Following PRISMA guidelines, 59 cohort studies published between 2014 and 2024 were analysed. Findings reveal mixed demographic patterns, with a slight male predominance in overall NEN incidence but site-specific variations influenced by genetic, hormonal, and environmental factors. Median age at diagnosis is 60 years, though appendiceal NENs typically affect younger individuals. Racial disparities were noted, with higher incidence rates among Black populations in the USA, though findings varied across studies. Globally, the incidence of NENs has increased, with the most notable surge reported in the USA between 1973 and 2012. Similar upward trends were observed in Europe, Asia, and Australia, though to different extents. However, variations in data collection methods present significant challenges for cross-national comparisons. The review highlights the need for standardised methodologies and further research to address gaps in understanding NEN epidemiology in smaller nations and underrepresented regions.

## Introduction

The ever-growing knowledge over the past decades in the field of neuroendocrine neoplasms (NENs) has brought more recognition and understanding of these tumours. NEN can be described as a group of rare heterogeneous tumours originating from diffuse neuroendocrine cells throughout the body, having both morphologically and immunohistochemically distinguishable features (Fraenkel *et al.* 2014, Takayanagi *et al.* 2022). Despite being considered rare tumours, several reports based on cancer registry data (Wyld *et al.* 2019, Masui *et al.* 2020, Prakash *et al.* 2020, Chang *et al.* 2021, Michael *et al.* 2022, White *et al.* 2022, Grundmann *et al.* 2023, Liu *et al.* 2023, Thiis-Evensen & Boyar Cetinkaya 2023, Wu *et al.* 2023, Zheng *et al.* 2023, Chi *et al.* 2024) and specialised tertiary centres (Stensbøl *et al.* 2021, Čustović *et al.* 2021, Escobar *et al.* 2022) established that the incidence and prevalence of NENs continue to rise globally, with figures varying according to the site of origin and tumour grade. However, it remains unclear whether this represents a true increase in incidence or enhanced detection capabilities across various countries. Furthermore, there is limited understanding of whether these trends are also observed in smaller nations, highlighting the need for further investigation into these knowledge gaps.

This review aims to analyse and discuss recently published data on the worldwide epidemiology of NEN, specifically focusing on gastroenteropancreatic NEN (GEP-NEN) and bronchopulmonary NEN (BP-NEN), and explore potential trends in incidence rates according to demographics, primary sites, grade and stage. Furthermore, survival patterns will be explored to find any significant global differences between large and small nations.

## Methods

A thorough review of the literature was conducted following the Preferred Reporting Items for Systematic Reviews and Meta-Analyses (PRISMA) guidelines to address the previously discussed areas on NEN and identify any research gaps.

### Eligibility criteria

Utilising the population, exposure, comparison and outcome (PECO) strategy (Moher *et al.* 2015), this review sought to answer the following question: ‘in patients (P) with NEN (E), is there a rising incidence and variations in survival (O) across different countries globally (C)’?

Cross-sectional studies that evaluated the epidemiology and survival of all subtypes of GEP and bronchopulmonary NEN within a defined population, which reported incidence and survival rates, were included. Both cancer registry data and databases from tertiary centres were included, minimising the limitations inherent in each and providing a more comprehensive and accurate overview of the epidemiology of NEN.

Reviews, systematic reviews and meta-analyses were excluded but reviewed to retrieve additional eligible individual studies. Letters, abstracts, correspondence, case reports, editorials and the grey literature were excluded.

### Information sources and search strategy

The searches were conducted using PubMed, Google Scholar and ScienceDirect databases. The search strategy was elaborated based on the MeSH terms (medical subject headings) according to the PECO acronym. In the search strategy, the following combinations of keywords were applied: ‘neuroendocrine neoplasia’, ‘neuroendocrine tumours’ or ‘neuroendocrine tumours’, ‘gastroenteropancreatic’ or ‘GEP-NEN’, ‘bronchopulmonary’ or ‘lung’, ‘carcinoid’, ‘small cell lung’, ‘large cell lung’, ‘epidemiology’, ‘incidence’, ‘prevalence’ and ‘survival’. Only studies published between 2014 and 2024 in the English language were included. The searches were conducted in September 2024.

### Selection process

The studies identified in the searches were exported to the reference manager (Zotero®) to remove duplicates and analysed according to the inclusion and exclusion criteria in two phases. In phase 1, the studies were screened based on their titles and abstracts. In phase 2, the full-text versions of the studies selected in phase 1 were read and included according to the previously defined criteria.

### Data collection process

The following data were collected from individual studies: year of publication, author, research country, study design, population assessed (including number of participants and any exclusion criteria), methods used and the main results.

## Results

### Selection of the studies

The searches in the electronic databases identified 5,305 references and, after duplicate removal, 2,813 references were assessed in phase 1. After reading the titles and abstracts, 107 studies were selected to be read in full.

**Figure 1:**
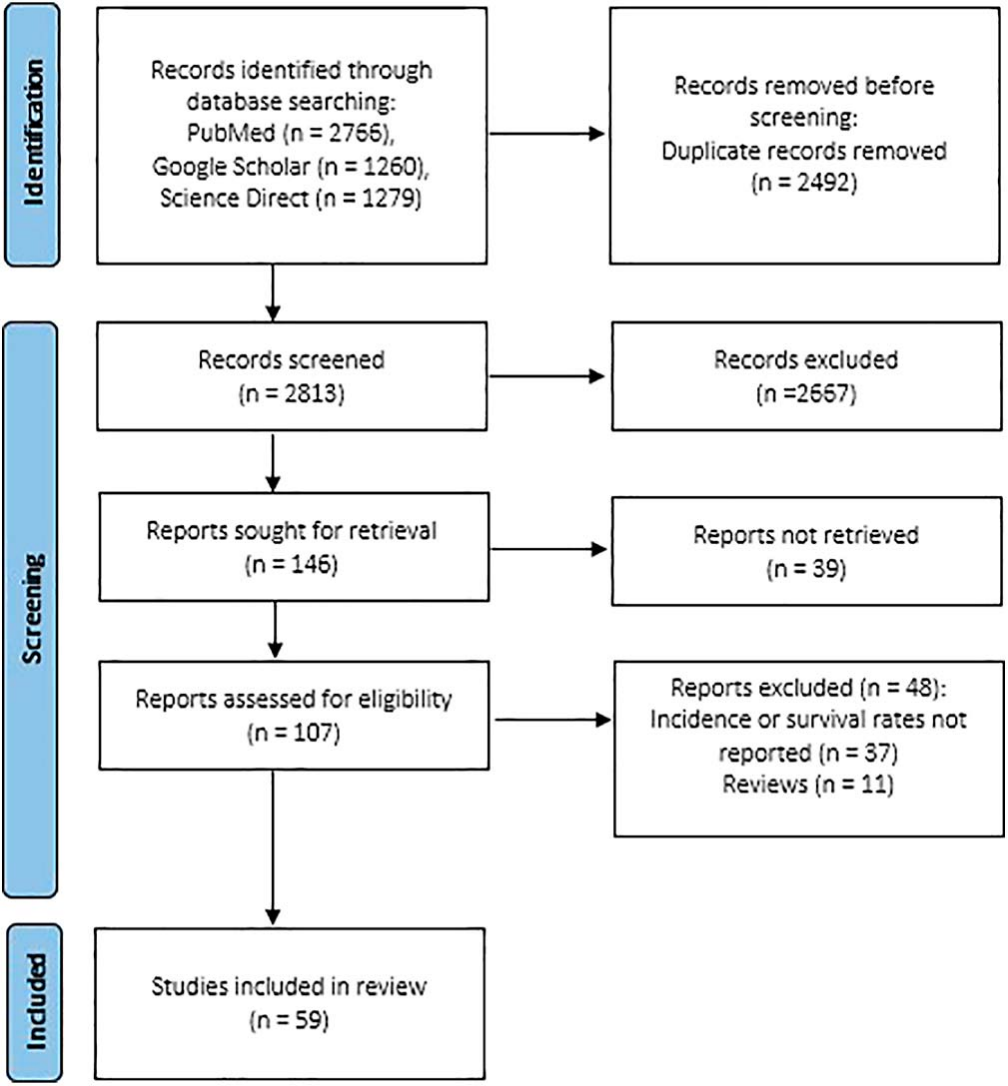
Flow diagram of the literature search and selection criteria.

In phase 2, 48 references were excluded and 59 studies were included in the final sample. The process of identification, inclusion and exclusion of studies is described in Fig. 1.

### Characteristics of the studies

The studies were published between 2014 and 2024 and conducted on four different continents. The majority (*n* = 28) focused on epidemiological findings or survival from America, followed by those from European countries (*n* = 20), Asia (*n* = 7), Australia and New Zealand (*n* = 4). All were cohort studies done retrospectively. Most used cancer-registry data (*n* = 48) while only 11 studies used data from specialised tertiary centres or compared cancer-registry data to histopathological reports. While some studies analysed NEN from all sites, others focused on a specific subtype.

### Global incidence and prevalence of NEN

The incidence and prevalence of NEN have been consistently reported to be increasing globally over the last decade. Most studies have used cancer registries and only a few have used data from tertiary hospitals. The most notable surge ever reported was in a large population-based study of 43,751 patients conducted in the United States (US), based on the National Cancer Institute’s Surveillance, Epidemiology and End Results (SEER) database, which covers only 30% of the US population (Dasari *et al.* 2017). Between 1973 and 2012, the annual age-adjusted incidence of all types of NEN increased by 6.4-fold, from 1.09 to 6.98 per 100,000 persons per year (Dasari *et al.* 2017). By 2018, this figure further increased to 8.19 per 100,000 persons per year, indicating a decrease in the annual percentage change per year over time (Wu *et al.* 2023). In a separate study done in Kentucky, the age-adjusted incidence rate of all NEN was reported at 10.3 per 100,000 persons per year (Chauhan *et al.* 2018). This suggests that SEER data may not fully represent the national incidence of NENs in the US and should be interpreted with caution. This is especially relevant for areas not captured by SEER where differences in population demographics and healthcare access may influence detection rates, leading to under- or overestimation of the true national burden. In Ontario, Canada, although the last published study reporting incidence data was published in 2015 by Hallett *et al.* an upward trend was also noted, with the standardised incidence rate (SIR) of all NEN in 2009 reported at 5.86 per 100,000 persons per year from 2.48 per 100,000 persons per year in 1994 (Hallet *et al.* 2015).

Similar to the US, England has reported a SIR of 8.8 per 100,000 persons in 2018, a four-fold increase from 2.5 per 100,000 persons in 1995 (White *et al.* 2022). Meanwhile, in Norway, a slightly higher incidence of 9.97 per 100,000 persons per year was observed in the period between 2017 and 2021, three times more than that reported between 1993 and 1996 (Thiis-Evensen & Boyar Cetinkaya 2023). This study included cases of phaeochromocytomas and medullary thyroid carcinomas, potentially contributing to the elevated incidence rates alongside the more recent data collection period. Consequently, discrepancies in data collection methodologies among different studies and registries may influence the reliability of cross-national comparisons. A study from a tertiary hospital in Bosnia and Herzegovina reported a much lower incidence rate, although not age-standardised, of 0.23 per 100,000 persons in 2020, highlighting how the disparities in data collection approaches may influence the results (Čustović *et al.* 2021).

Although epidemiological studies on the incidence of NENs in Australia are limited, two studies, one held in Victoria (Michael *et al.* 2022) and the other in Queensland (Wyld *et al.* 2019), also mirror the results reported elsewhere. A three-fold increase in the age-adjusted incidence was noted during their respective study periods, with an incidence rate of 9.7 per 100,000 persons per year in 2019 in Victoria (Michael *et al.* 2022) and 6.3 per 100,000 per year in 2015 in Queensland (Wyld *et al.* 2019). Notably, the Victoria study excluded small-cell and large-cell lung carcinomas as well as lung neuroendocrine carcinoma (NEC), while the Queensland study did not include NEC and Merkel cell carcinoma. This selective exclusion may lead to an underestimation of the overall incidence of NENs in these states.

In Asia, despite a similar notable rising trend, the overall age-adjusted SIR remains lower than that observed in the other continents. Registry-based studies done in Singapore and Taiwan reported an incidence of 2.4 per 100,000 persons per year in 2014 (Prakash *et al.* 2020) and 3.16 per 100,000 persons per year in 2015, respectively (Chang *et al.* 2021). However, findings from China present some discrepancies; while the age-adjusted SIR of all NEN in a national study was reported at 1.14 per 100,000 persons per year in 2017 (Zheng *et al.* 2023), a separate study reported a higher incidence of 3.58 per 100,000 persons per year in 2018 in Beijing (Chi *et al.* 2024). The latter study included cases of small cell and large cell lung carcinoma, which may have contributed to a more comprehensive view of NEN incidence in a sub-cohort of China.

The rise in the prevalence of NEN aligns with the increasing incidence observed. In the US, the 20-year limited-duration prevalence rates significantly increased, from 3.8 per 100,000 persons in 1998 to 60 per 100,000 persons in 2018 (Wu *et al.* 2023). Norway has reported a more marked increase, which may be attributed to differences in inclusion criteria, with NEN prevalence rising from 18.2 per 100,000 persons in 1993 to 120.9 per 100,000 persons by 2021 (Thiis-Evensen & Boyar Cetinkaya 2023). However, comprehensive prevalence data from Europe, Asia or Australia remain insufficient in the existing literature.

The global rise in the incidence and prevalence of NEN can be attributed to multiple factors. Over time, there has been improved awareness among physicians, advancements in diagnostic screening tools including enhanced imaging techniques and improved healthcare resources that have facilitated the detection of more incidental NENs during investigations for unrelated health conditions. This increase in detection rates may partially account for the global surge in NEN incidence (Prakash *et al.* 2020, Chang *et al.* 2021, Michael *et al.* 2022, Thiis-Evensen & Boyar Cetinkaya 2023). Furthermore, the publication of updated histopathological NEN classifications has appropriately reclassified tumours previously known as undifferentiated into the NEN category. The World Health Organisation (WHO) classification, recently updated in 2022, is considered the gold standard for diagnosing this disease. A significant revision in the classification occurred with the 2019 WHO update, which introduced a new category for well-differentiated G3 neuroendocrine tumours, while NEC were classified as a separate entity without a grade and divided into small cell and large cell NEC (Assarzadegan & Montgomery 2021). Variations in reported incidence rates across different countries, however, may be attributed to the implementation of different versions of the International Classification of Diseases for Oncology (ICD-O) coding system and the WHO classifications, resulting in early reports often being inconsistent with more recent data. In addition, relying solely on pathological diagnoses, common in cancer registry-based studies, may underestimate the actual incidence, excluding cases diagnosed radiologically (Box 1). Hence, further research using a triangulation of data collection methods and standardised diagnostic classifications is necessary to achieve a comprehensive understanding of the global incidence and prevalence of NEN.

Box 1 **Potential contributing factors to the reported discrepancies in the incidence of NEN globally**

- Retrospective nature of the studies.
- Different inclusion criteria.
- Different WHO classifications used.
- Lack of standardised pathological reporting.
- Data registry variations.
- Access to healthcare resources.
- Access to advanced diagnostic techniques.
- Socioeconomic status.
- Genetic and racial influence.
- Environmental exposures.
- Lifestyle factors (e.g. smoking, alcohol, diet).

### Incidence of NEN according to primary site

The epidemiological studies on NEN present a mixed picture regarding sex predominance, primary tumour site predilection, age at diagnosis and racial/ethnic disparities, with no consistent trends between large and small nations. While the majority report a slight male predominance in the incidence of NEN (Patel & Benipal 2019, Chang *et al.* 2021, Čustović *et al.* 2021, Borbath *et al.* 2022, Zheng *et al.* 2023, Chi *et al.* 2024), few studies challenge these findings and report either no significant sex difference (Prakash *et al.* 2020, Xu *et al.* 2021, White *et al.* 2022) or even a slight female predominance (Gudmundsdottir *et al.* 2019, Wyld *et al.* 2019, Alwan *et al.* 2020, Wu *et al.* 2023).

NEN can develop at any anatomical site, most commonly occurring in the bronchopulmonary and GEP regions (Wu *et al.* 2023, Chi *et al.* 2024). The global incidence of GEP-NEN, as reported by cancer registry-based studies, is relatively similar across different countries. In Bavaria, Germany, it was reported at 4.8 per 100,000 persons per year in 2019 (Grundmann *et al.* 2023), while Switzerland recorded a rate of 4.18 per 100,000 persons per year in 2016 (Alwan *et al.* 2020). In the US, the incidence was noted at 4.18 per 100,000 persons per year in 2018 (Wu *et al.* 2023), and Japan reported a lower rate of 3.53 per 100,000 persons per year in 2016 (Masui *et al.* 2020). In contrast, a study done in South-Western Norway, which included patients diagnosed with GEP-NEN through histological or radiological methods, reported a SIR of 6.62 per 100,000 persons per year in 1993, highlighting the discrepancy between cancer registry-based studies and those done at tertiary medical centres (Sandvik *et al.* 2016). This is further highlighted in another study done in a tertiary hospital in Seoul, Japan, which reported a SIR of GEP-NEN of 24.1 per 100,000 persons per year (Lim *et al.* 2017).

Few studies specifically aim to evaluate trends in the epidemiology of bronchopulmonary NEN. Most report the incidence as part of a broader evaluation of different types of NEN. The incidence rates of BP-NEN are comparable in the US (Dasari *et al.* 2017, Broder *et al.* 2018, Wu *et al.* 2023), England (White *et al.* 2022) and Australia (Michael *et al.* 2022), ranging between 1.3 and 1.64 per 100,000 persons per year. Conversely, a higher incidence was noted in Beijing, China at 2.38 per 100,000 persons per year in 2018 (Chi *et al.* 2024) and in Switzerland, with an average SIR of 6.2–9.2 per 100,000 persons per year (Alwan *et al.* 2020). Notably, the latter two studies included lung carcinoid, small cell and large cell lung carcinoma, thus providing a more accurate representation of BP-NEN incidence in these populations. Analysis of the different subtypes of BP-NEN showed that the SIR of small cell lung cancer declined over the years, while that of large cell lung cancer doubled from the initial reports (Shah *et al.* 2021). An upward trend in the incidence was also noted in typical and atypical carcinoids, with Petursdottir *et al.* (2020) reporting a triple increase in the incidence of pulmonary carcinoids over 6 decades in the Icelandic population (Petursdottir *et al.* 2020). Supplementary Table 1 (see section on Supplementary materials given at the end of the article) summarises population-based studies published between 2014 and 2024 reporting the SIR of NEN based on different primary sites.

Racial and ethnic disparities in NEN incidence have also been reported, though findings are inconsistent across studies. Using the SEER database and the Medicare claims data, Shen *et al.* (2019) observed a higher incidence of NEN in the Black population compared to the White population, with rates of 7.3 per 100,000 persons per year for Blacks in contrast to 5.37 per 100,000 persons per year in non-Hispanic Whites and 4.18 per 100,000 persons per year in Hispanic Whites (Shen *et al.* 2019). Similarly, this was observed by Patel & Benipal (2019), who analysed data from the US Cancer Statistics, which integrates information from both the Centres for Disease Control and Prevention’s (CDC) National Program of Cancer Registries (NPCR) and the SEER database, and hence offers a comprehensive representation of the US population (Patel & Benipal 2019).

However, differing findings emerge from other studies reporting a higher incidence of NEN in the White population when compared to Blacks, Asians or Pacific Islanders (Xu *et al.* 2021). Furthermore, Whites are more likely to present with more aggressive tumour types, exhibiting the highest overall incidence of high-grade NENs in the US at 11.02 per 100,000 persons per year between 2010 and 2012 (Leoncini *et al.* 2017). This further supports previous findings by Shen *et al.* (2019), where poorly differentiated and undifferentiated NEN were more commonly found in Hispanic Whites (26.33%) compared to non-Hispanic Whites (23.58%) and Blacks (22.85%) (Shen *et al.* 2019). However, of note, these findings are primarily derived from US-based studies with limited evidence from small nations. While biological and genetic differences may contribute to these racial and geographic disparities, there is a lack of comprehensive data regarding patients’ socio-economic status and healthcare access, factors that could also contribute to the observed inconsistencies across studies.

Most studies indicate that males are more commonly affected by colonic, pancreatic, rectal, small intestinal and gastric NENs, while females are more frequently affected by appendiceal and lung NENs (Alwan *et al.* 2020, White *et al.* 2022, Grundmann *et al.* 2023). These site-specific differences are poorly understood and may involve a complex interplay of genetic, hormonal and environmental factors. In addition, males were found to be more susceptible to developing high-grade tumours (Leoncini *et al.* 2017, Jann *et al.* 2023), with a reported male-to-female ratio of 1.2 (Leoncini *et al.* 2017).

The median age at diagnosis across all sites is 60 years (IQR 18–102) (Borbath *et al.* 2022). However, appendiceal NENs tend to affect individuals younger than 40 years old (Ribeiro *et al.* 2021, White *et al.* 2022, Grundmann *et al.* 2023). While patients older than 50 years had a higher absolute incidence rate of GEP-NEN (Dasari *et al.* 2017, Lee *et al.* 2019), younger individuals experienced a higher rate of increase in incidence over 43 years, between 1975 and 2018. This suggests that the epidemiology of NEN may be changing, possibly due to variations in risk factors or improved diagnostic practices (Yao *et al.* 2023).

**Figure 2:**
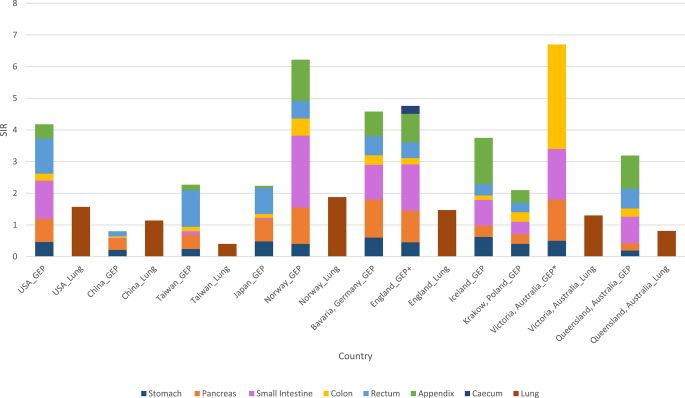
The global distribution of gastroenteropancreatic (GEP) NEN and lung NEN based on SIR.

The distribution of NEN based on primary site also differs across racial, ethnic and geographic regions (Shen *et al.* 2019) (Fig. 2). BP-NENs are common among non-Hispanic White Americans (Shen *et al.* 2019), Europeans (White *et al.* 2022) and Asians (Chi *et al.* 2024), with non-Hispanic Whites exhibiting the highest incidence across all stages in a comparative study involving non-Hispanic Whites, Hispanic Whites, Blacks and other races in the SEER dataset (Shen *et al.* 2019).

Rectal NENs are more frequently observed in Asians (Lim *et al.* 2017, Masui *et al.* 2020, Chang *et al.* 2021, Wu *et al.* 2023, Chi *et al.* 2024), African Americans (Shen *et al.* 2019) and Australians (Michael *et al.* 2022). Conversely, Europeans show a much lower incidence of rectal NENs, with rates of 0.37–0.6 per 100,000 persons per year (Gudmundsdottir *et al.* 2019, White *et al.* 2022, Grundmann *et al.* 2023), compared to 1.15–1.82 per 100,000 persons per year in Asians (Masui *et al.* 2020, Chang *et al.* 2021). However, the pancreas is a common primary site for GEP-NEN among both Asians (Ito *et al.* 2015, Masui *et al.* 2020, Chang *et al.* 2021, Wu *et al.* 2023, Zheng *et al.* 2023) and Europeans (White *et al.* 2022, Grundmann *et al.* 2023, Thiis-Evensen & Boyar Cetinkaya 2023) despite a lower incidence reported in Asians.

Small intestinal NENs are more commonly seen in African Americans (Shen *et al.* 2019, Chang *et al.* 2021), Europeans (Trofimiuk-Müldner *et al.* 2017, Stensbøl *et al.* 2021, Borbath *et al.* 2022, Snorradottir *et al.* 2022, White *et al.* 2022, Grundmann *et al.* 2023) and Australians (Wyld *et al.* 2019, Michael *et al.* 2022), with a significantly higher incidence difference observed between Blacks and Whites (22 vs 13%, *P* < 0.001) (DePalo *et al.* 2019). In a study on small intestinal tumours, jejunoileal NEN were found to be the most common subtype of small intestinal cancer compared to jejunoileal adenocarcinoma, duodenal NEN and duodenal adenocarcinoma, with an age-adjusted SIR of 8.07 per 100,000 per year (Landerholm 2021). Moreover, the incidence of appendiceal NEN appears to be higher in Europe and Australia, with a SIR of 0.78–1.3 per 100,000 persons per year in Europe (Genus *et al.* 2019, Gudmundsdottir *et al.* 2019, Ribeiro *et al.* 2021, White *et al.* 2022, Grundmann *et al.* 2023, Thiis-Evensen & Boyar Cetinkaya 2023) and 1.04 per 100,000 persons per year in Queensland, Australia (Wyld *et al.* 2019). However, studies from China do not report the incidence of appendiceal NEN (Zheng *et al.* 2023, Chi *et al.* 2024), and while the reason was not explicitly explained, it is possible these were also combined with colorectal tumours.

The global incidence of the least frequently documented NEN, gastric NEN, is similar between different countries. Most studies report a SIR between 0.19 and 0.92 per 100,000 persons per year. However, a study by Yang *et al.* (2018), which analysed gastric NEN cases from the SEER database, reported a higher incidence of 4.85 per 100,000 persons per year in 2014. This discrepancy may be an overestimation, potentially attributable to the absence of standardised classification criteria for case selection and the lack of a centralised pathology review (Yang *et al.* 2018).

The increasing incidence of NENs is notable across all primary sites, though the extent varies by country. Most studies observed a higher average annual growth in rate for pancreatic, rectal and gastric NENs (Wyld *et al.* 2019, Chang *et al.* 2021, Michael *et al.* 2022, White *et al.* 2022, Grundmann *et al.* 2023). The incidence rate of rectal NEN has significantly increased in younger adults; however, this has remained stable in older adults (*P* < 0.001) (Abboud *et al.* 2023). Other studies have also reported the appendix and small intestine as sites with the highest increase in the incidence rate of NEN (Patel & Benipal 2019, Grundmann *et al.* 2023, Liu *et al.* 2023, Thiis-Evensen & Boyar Cetinkaya 2023, Wu *et al.* 2023).

Furthermore, differences in NEN incidence between urban and rural populations have been noted in some studies, with rural areas exhibiting a higher incidence rate than urban settings (Hallet *et al.* 2015, Gosain *et al.* 2019). This disparity may also highlight that potential exposure to environmental endocrine-disrupting chemicals could induce oncogenic genetic modulation, contributing to the increased incidence observed in rural populations.

### Incidence of NEN according to grade and stage

A consistent rise in NEN incidence has been reported across all grades and stages (Dasari *et al.* 2017). In the USA and Germany, the most pronounced increases were seen in localised tumours and grade 1 (G1) NEN (Patel & Benipal 2019, Grundmann *et al.* 2023, Wu *et al.* 2023), while in Victoria, Australia, grade 2 (G2) and grade 3 (G3) tumours had the greatest increase (Michael *et al.* 2022). This latter trend could be attributed to changes in histological classification during the study period. In Europe, 47% of patients with NEN had metastatic disease at the time of diagnosis (Borbath *et al.* 2022). However, the proportion of patients presenting with metastatic disease has remained constant over the years despite a slight increase in incidence, hinting at the possibility that improved diagnostic techniques rather than an actual rise in epidemiology may be at play (Wu *et al.* 2023).

While improved diagnostic endoscopic procedures and enhanced screening programmes likely account for these increases, it remains uncertain whether this reflects a true rise in incidence or merely improved detection of pre-existing tumours. As a result, this complicates our understanding of NEN epidemiology and necessitates cautious interpretation of data.

### Mortality and survival patterns

Although an increase in mortality rates was observed, which broadly reflected the rise in the incidence rates, this rise in rates was not to the same degree. Reports on incidence-based mortality rates are limited, but Wu *et al.* (2023) reported an incidence-based mortality rate of 2.31 per 100,000 persons annually in 2018, which increased from 0.74 in 2000 in the USA, reflecting an annual percentage change of 4.14 (95% CI, 3.14–5.15). This rise in mortality rate was observed in all stages and grades of disease, with localised and G1 disease showing the most significant increases. The highest incidence-based mortality rates were observed in patients with lung, small intestine and pancreas at rates of 0.57, 0.24 and 0.23 per 100,000 persons per year, respectively (Wu *et al.* 2023). In addition, patients diagnosed with early onset GEP-NEN before the age of 50, apart from showing a higher rise in incidence when compared to those with a later onset of diagnosis, also exhibited a more significant increase in mortality rates (Yao *et al.* 2023). This trend was particularly evident in high-grade (G3 and G4) and distant disease (Yao *et al.* 2023), suggesting that advanced disease may be more aggressive in younger patients.

Survival rates for NENs have also improved, although disparities among various regions persist. Queensland, Australia reported the highest 5-year relative survival rate (RSR) at 92% from 2005 to 2015 (Wyld *et al.* 2019). In the US, the 5 and 10-year RSR for all NEN were 68.4 and 63.5%, respectively, during the period from 2000 to 2018 (Wu *et al.* 2023). Notably, as most US data is derived from the SEER database, reported survival rates may be higher than those observed in regions with more limited resources. In Europe, these rates were similarly recorded at 74.5 and 61% (Borbath *et al.* 2022). However, survival rates in Asia were notably lower, with China showing overall survival (OS) rates of 26.3% and 23.1% for 5 and 10 years (Chi *et al.* 2024), respectively, while Singapore’s rates were recorded at 38.1 and 22.0% (Prakash *et al.* 2020).

Specific survival rates vary significantly based on factors such as age, primary tumour site, tumour grade and stage at diagnosis (Hallet *et al.* 2015, Dasari *et al.* 2017, Reeders *et al.* 2019, Xu *et al.* 2021, Borbath *et al.* 2022, White *et al.* 2022) which may partly explain the variations in survival outcomes across different regions. Older age, male sex, higher grades and more advanced disease stages were all associated with worse prognoses (Genc *et al.* 2018, Wyld *et al.* 2019, Chang *et al.* 2021, Borbath *et al.* 2022, Escobar *et al.* 2022, White *et al.* 2022, Wu *et al.* 2023). In a large Dutch cohort study by Poleé *et al.* (2022), the 5-year OS and RSR for non-metastatic GEP-NENs were 81 and 88% for grade 1, 78 and 83% for grade 2, and 26 and 30% for grade 3 tumours, respectively. For metastatic GEP-NEN, the corresponding 5-year OS and RSR were 47 and 52% for grade 1, 38 and 41% for grade 2, and 5 and 5% for grade 3 tumours (Poleé *et al.* 2022).

Patients with low to intermediate aggressive disease had a better 5-year RSR when compared to those with more aggressive NEN, with rates of 64.8 and 8.4% respectively (Boyar Cetinkaya *et al.* 2018). In a study evaluating poorly differentiated lung and extrapulmonary NEC, the 5-years OS was found to be lowest for lung NECs at 5.6%, followed by gastrointestinal NEC at 13.1% and NECs located at other primary sites at 26.0%. Notably, small intestinal NEC demonstrated a more favourable 5-year OS rate of 34.8%. However, these studies classified patients based on tumour morphology and behaviour rather than the proliferation index Ki67 or the WHO grade, limiting the overall interpretation of the results (Dasari *et al.* 2018).

In addition, some studies indicate that race is an independent predictor for survival, with Black, American Indian and Alaska Native populations experiencing worse outcomes (Shen *et al.* 2019, Wu *et al.* 2023). In a study evaluating pancreatic NEN, it was reported that Black patients had a lower OS rate when compared to non-Black patients (HR = 1.17, 95% CI: 1.00–1.37, *P* = 0.046) and worse pancreatic neuroendocrine tumour-specific survival (HR = 1.22, 95% CI: 1.01–1.48, *P* = 0.044) (Zhou *et al.* 2017). Nonetheless, Shah *et al.* (2021) observed that race did not impact survival in lung NEN (Shah *et al.* 2021).

Most studies observed the highest RSR in tumours located in the rectum, appendix and small intestine (Man *et al.* 2018, Wyld *et al.* 2019, Chang *et al.* 2021, Escobar *et al.* 2022, Poleé *et al.* 2022, White *et al.* 2022, Wu *et al.* 2023) while tumours in the pancreas, colon, lung, oesophagus and biliary tract present the least favourable prognosis (Boyar Cetinkaya *et al.* 2018, Gudmundsdottir *et al.* 2019, Wyld *et al.* 2019, Chang *et al.* 2021, Borbath *et al.* 2022, Escobar *et al.* 2022, Wu *et al.* 2023, Zheng *et al.* 2023).

Over time, survival has improved across all sites to different extents, with significant improvement noted in RSR for pancreatic tumours (Chang *et al.* 2021, Sonbol *et al.* 2022, White *et al.* 2022, Wu *et al.* 2023) where 5 and 10-year RSR reached 58.9 and 45.9%, respectively in the United States by 2013 (Wu *et al.* 2023). Similarly, in the Netherlands, the overall 5-years survival was 53% (Genc *et al.* 2018). Early detection may contribute to these outcomes; however, advancements in systemic treatment are also likely to contribute. Nonetheless, the association between treatment and survival outcomes cannot be fully ascertained due to the lack of detailed treatment information in most registry-based studies.

## Conclusion

The global rise in the incidence of NENs presents a significant public health challenge that demands a comprehensive, multifaceted approach. This review aimed to provide a comprehensive overview of the epidemiology of NENs and compare patterns between large and small nations using both cancer-registry data and databases from tertiary centres. While cancer-registry data are useful to identify demographic patterns, geographic distributions and changes in disease burden over time, they may lack clinical details and essential histopathological details due to coding limitations and underdiagnosis. Databases from tertiary or referral centres, despite possibly bearing a degree of referral bias, often include more detailed clinical and histopathological information, providing deeper insights into disease classification, staging, treatment patterns and outcomes. By incorporating both data sources, this review enhances the reliability and generalisability of findings.

Although published studies reveal some trends suggesting differences in incidence and survival of NENs between large and small nations, the evidence remains insufficient to draw firm conclusions due to significant data gaps and inconsistencies. Variations in classification systems, inclusion criteria and reporting standards hinder meaningful cross-country comparisons. To address this, future research should prioritise the development and adoption of standardised diagnostic and reporting pathology assessments to be implemented worldwide, facilitating more accurate and comparable data collection.

Furthermore, the limited comprehensive data from smaller nations highlights a critical need to expand cancer registries in underrepresented regions and to implement advanced diagnostic tools that improve case detection and classification. Nonetheless, the challenges posed by global migration continue to complicate epidemiological studies by introducing diverse ethnicities, lifestyle, environmental exposures and socioeconomic factors that can affect disease incidence and outcomes. Thus, future research should also prioritise stratifying data collection by ethnicity and socioeconomic status to address these disparities effectively and deepen our understanding of the disparities in NEN epidemiology between large and small nations.

## Supplementary materials



## Declaration of interest

The authors declare that there is no conflict of interest that could be perceived as prejudicing the impartiality of the work reported.

## Funding

This work did not receive any specific grant from any funding agency in the public, commercial or not-for-profit sector.

## Author contribution statement

Dr Mangion contributed to the idea for the article. Dr Mangion and Prof. Gruppetta performed the search strategy, selection process and data collection. The manuscript was written by Dr Mangion. Prof. Gruppetta and Prof. Vassallo have critically revised the work. All authors approved the final manuscript.
